# Supplementary material for: Dipicolinate Complexes of Oxovanadium(IV) and Dioxovanadium(V) with 2-Phenylpyridine and 4,4′-Dimethoxy-2,2′-bipyridyl as New Precatalysts for Olefin Oligomerization
Source: Materials (Basel). 2022 Feb 13;15(4):1379. doi: 10.3390/ma15041379 (PMC8875215; doi:10.3390/ma15041379)
Supplement: Supplementary file 1 [file materials-15-01379-s001.zip › materials-1580885-supplementary.pdf]

SUPPLEMENTARY MATERIAL

Dipicolinate complexes of oxovanadium(IV) and dioxovanadium(V) with 2-phenylpyridine and 4,4'-dimethoxy-2,2'-bipyridyl as new precatalysts for olefin oligomerization

Joanna Drzeżdżon[1]*, Marta Pawlak[1], Barbara Gawdzik[2], Aleksandra Wypych[3], Karol Kramkowski[4], Paweł Kowalczyk[5]*, Dagmara Jacewicz[1]

[1]Department of Environmental Technology, Faculty of Chemistry, University of Gdansk, Wita Stwosza 63, 80-308 Gdansk, Poland; joanna.drzezdzon@ug.edu.pl, marta.pawlak0812@gmail.com, dagmara.jacewicz@ug.edu.pl,

[2] Institute of Chemistry, Jan Kochanowski University, Uniwersytecka 7, 25406, Kielce, Poland, b.gawdzik@ujk.edu.pl

[3]Centre for Modern Interdisciplinary Technologies Nicolaus Copernicus University in Torun, Wileńska 4, 87-100 Toruń, Poland, wypych@umk.pl

[4]Department of Physical Chemistry, Medical University of Bialystok, Kilińskiego 1,15-089 Białystok, Poland; kkramk@wp.pl

[5]Department of Animal Nutrition, The Kielanowski Institute of Animal Physiology and Nutrition, Polish Academy of Sciences, Instytucka 3, 05-110 Jabłonna, Poland; p.kowalczyk@ifzz.pl

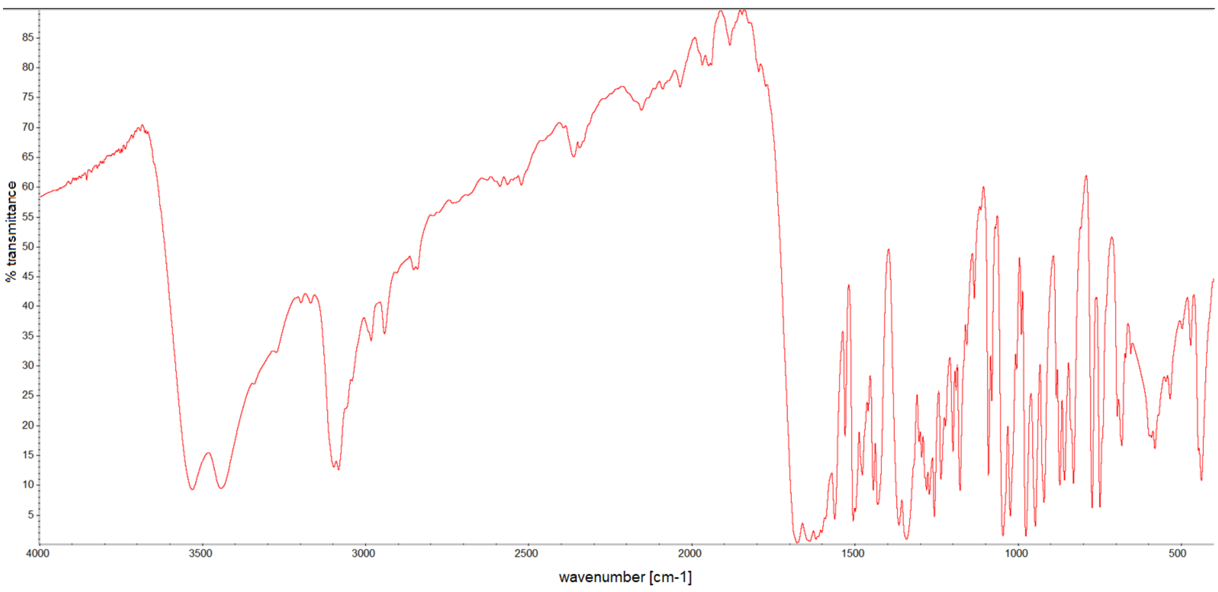

Figure S1. The IR spectra of [VO(dipic)(dmbipy)] · 2 H_2O

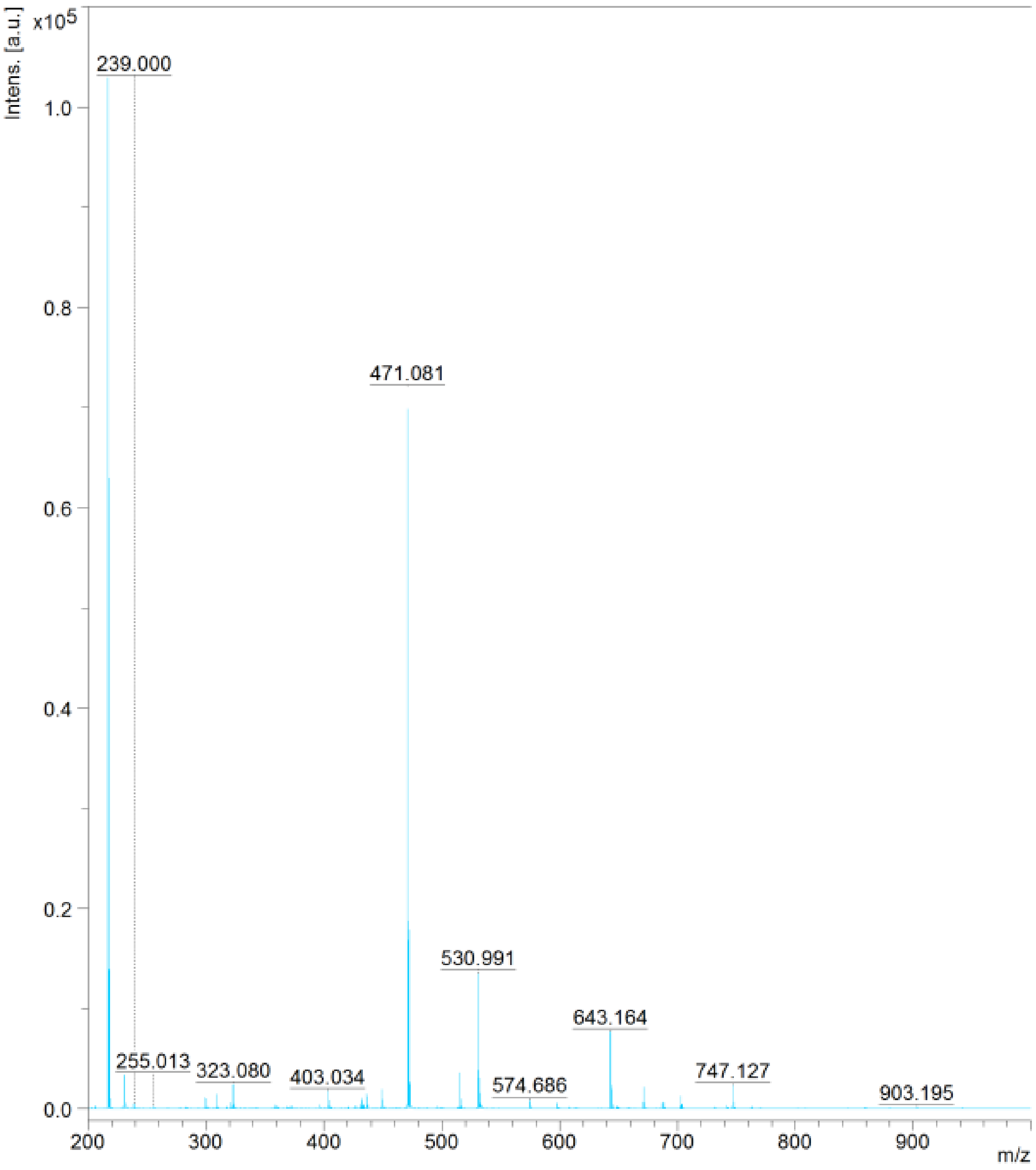

Figure S2. The MALDI-TOF-MS spectra of [VO(dipic)(dmbipy)] · 2 H_2O (CAA was a matrix)

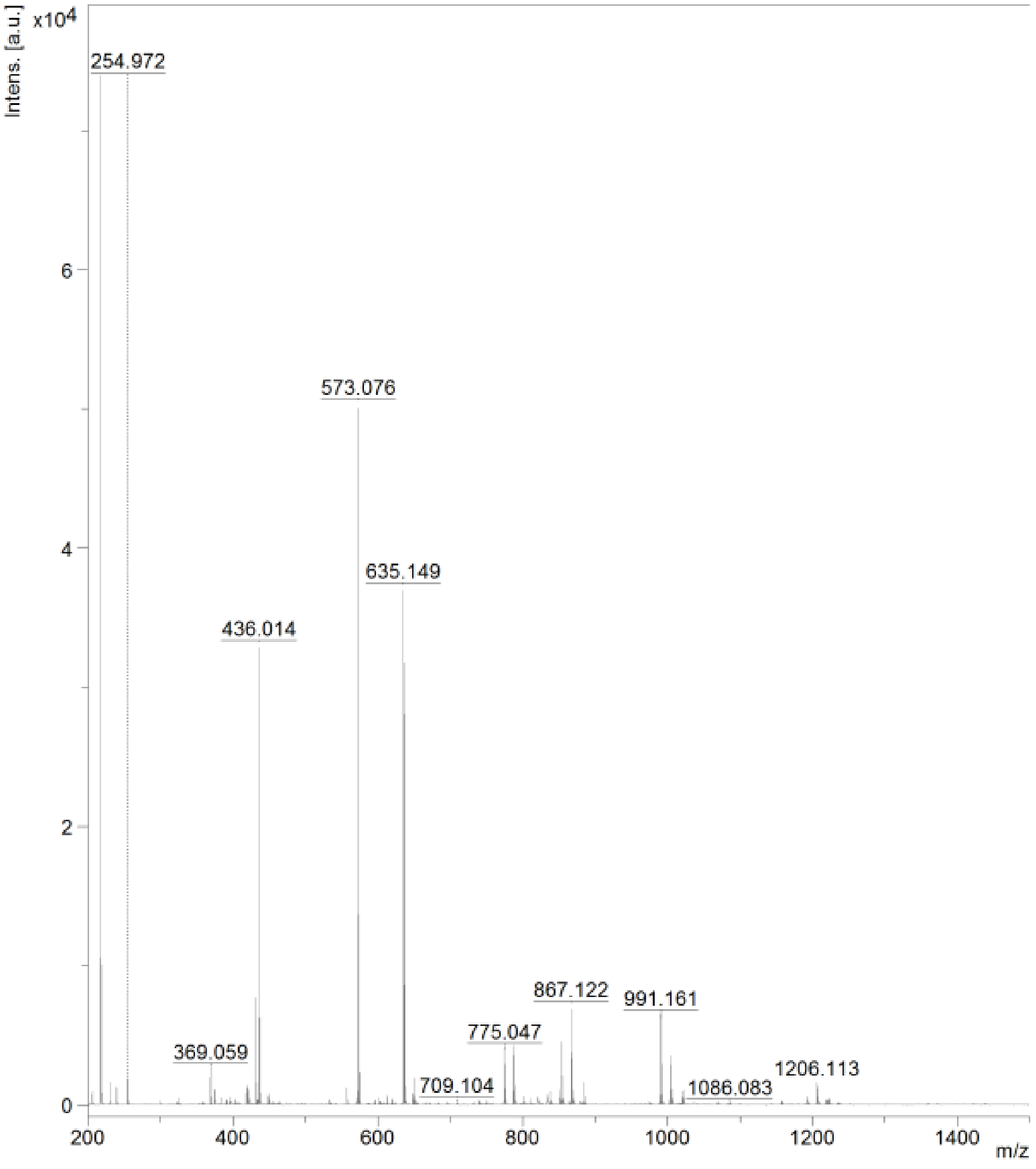

Figure S3. The MALDI-TOF-MS spectra of [VO(dipic)(dmbipy)] · 2 H_2O (DHB was a matrix)

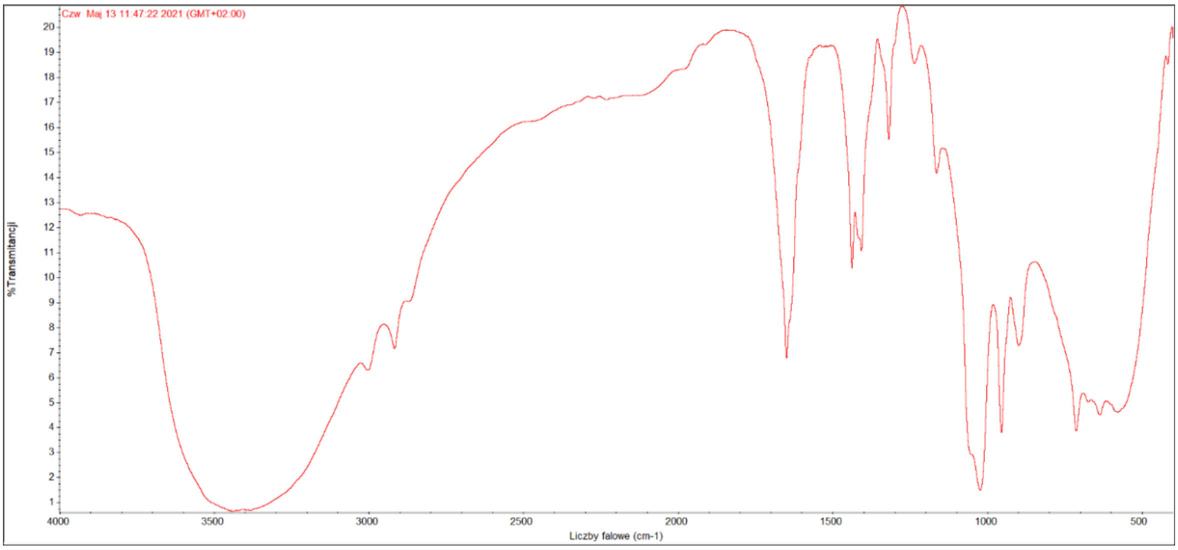

Figure S4. The IR spectra of 2-chloro-2-propen-1-ol oligomers obtained using [VOO(dipic)](2-phepyH) · H_2O

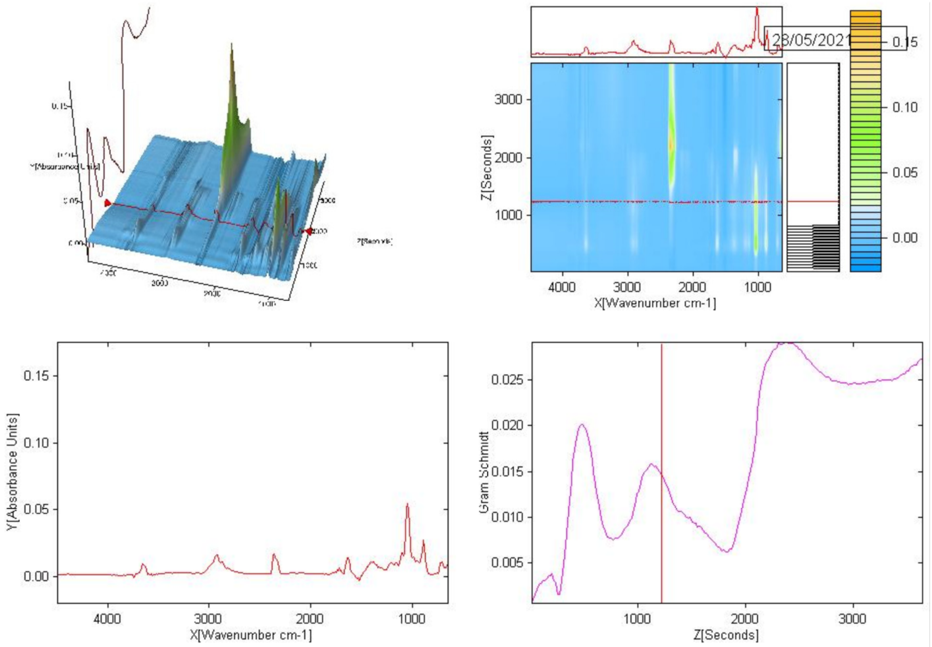

Figure S5. The TG-FTIR of 2-chloro-2-propen-1-ol oligomers obtained using [VOO(dipic)](2-phepyH) · H_2O

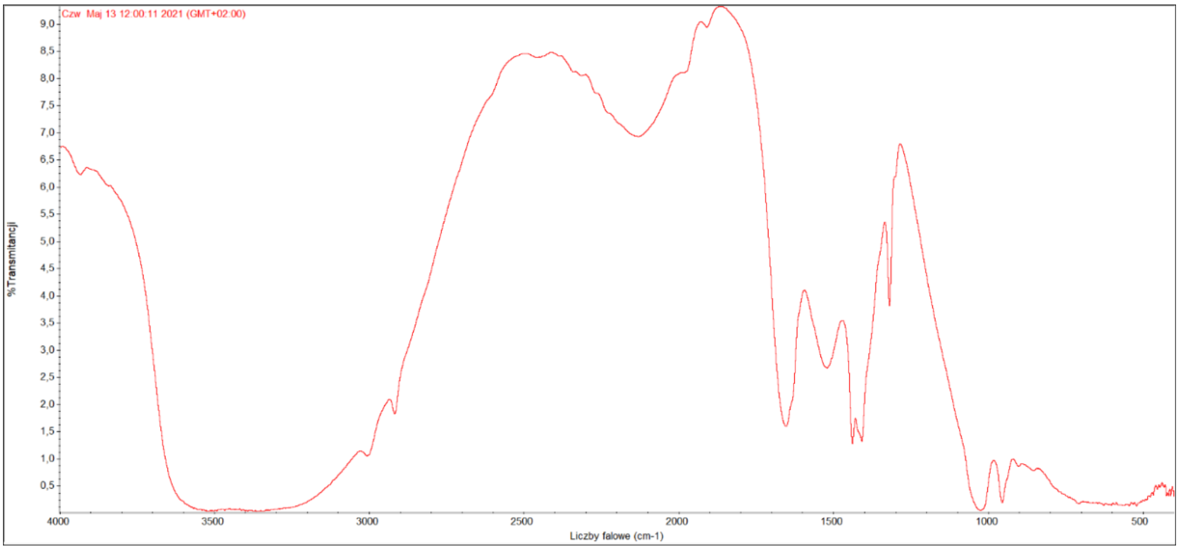

Figure S6. The IR spectra of norbornene oligomers obtained using [VOO(dipic)](2-phepyH) · H_2O

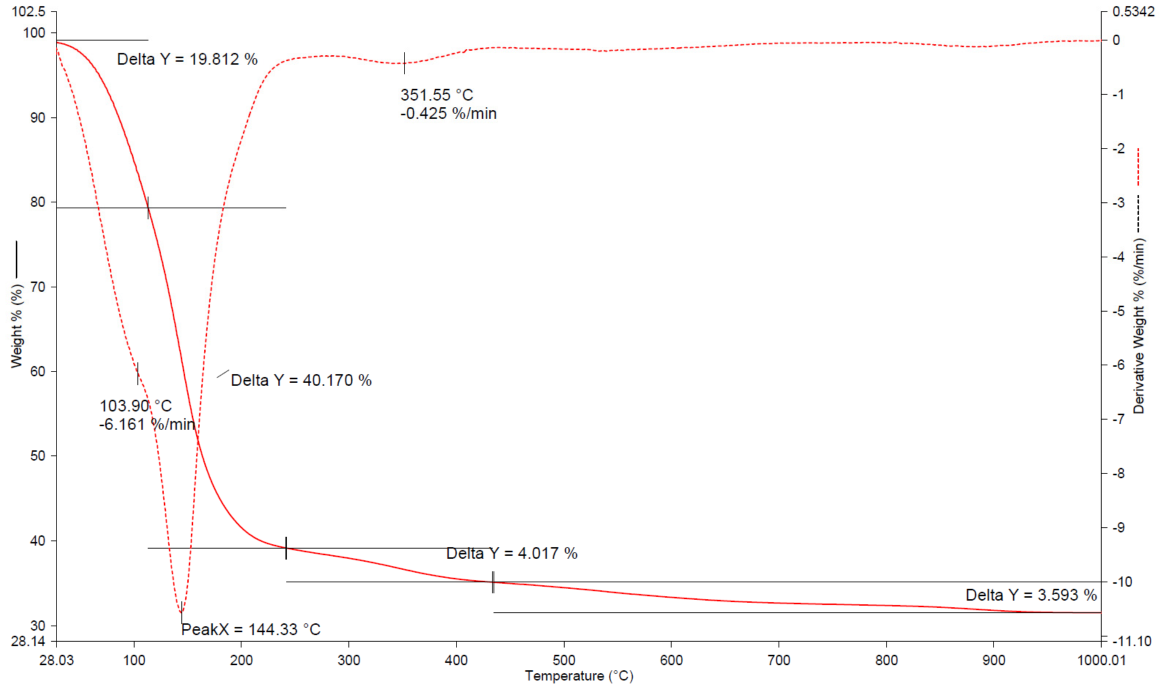

Figure S7. The TG of norbornene oligomers obtained using [VOO(dipic)](2-phepyH) · H_2O

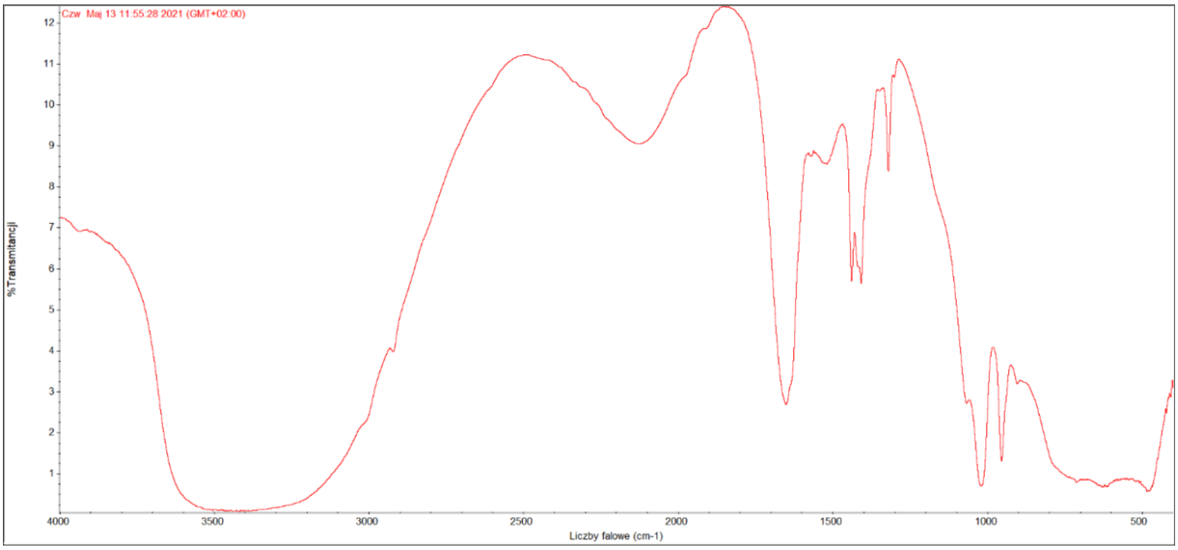

Figure S8. The IR spectra of 2-propen-1-ol oligomers obtained using [VOO(dipic)](2-phepyH) · H_2O

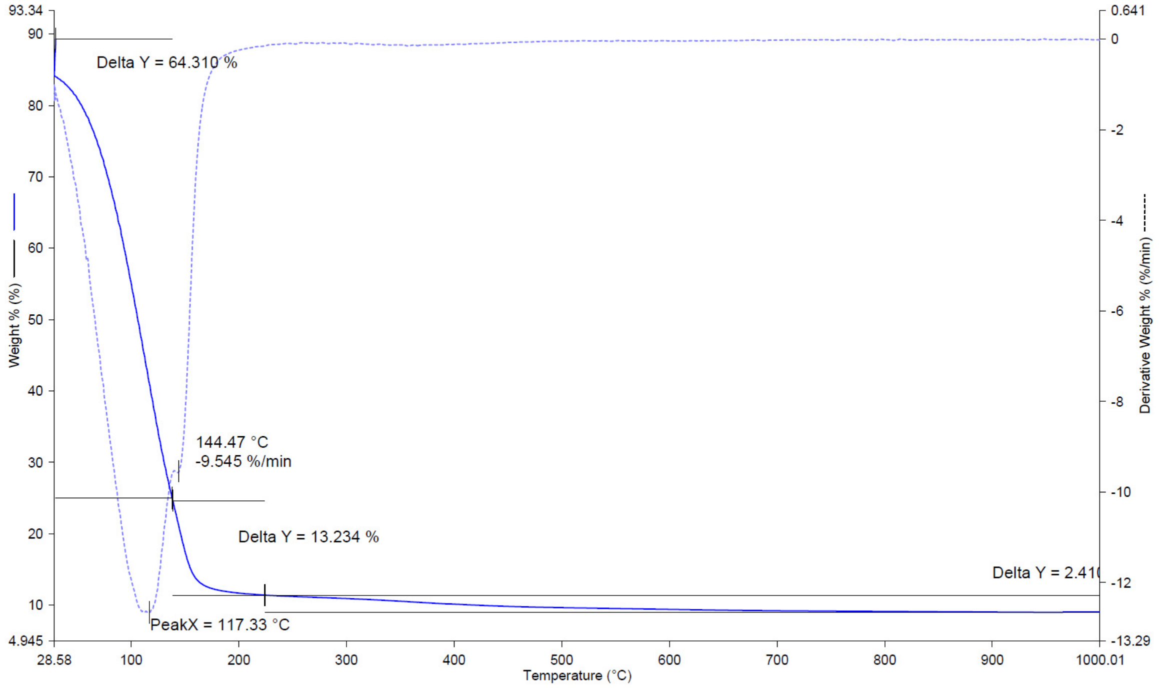

Figure S9. The TG of 2-propen-1-ol oligomers obtained using [VOO(dipic)](2-phepyH) · H_2O

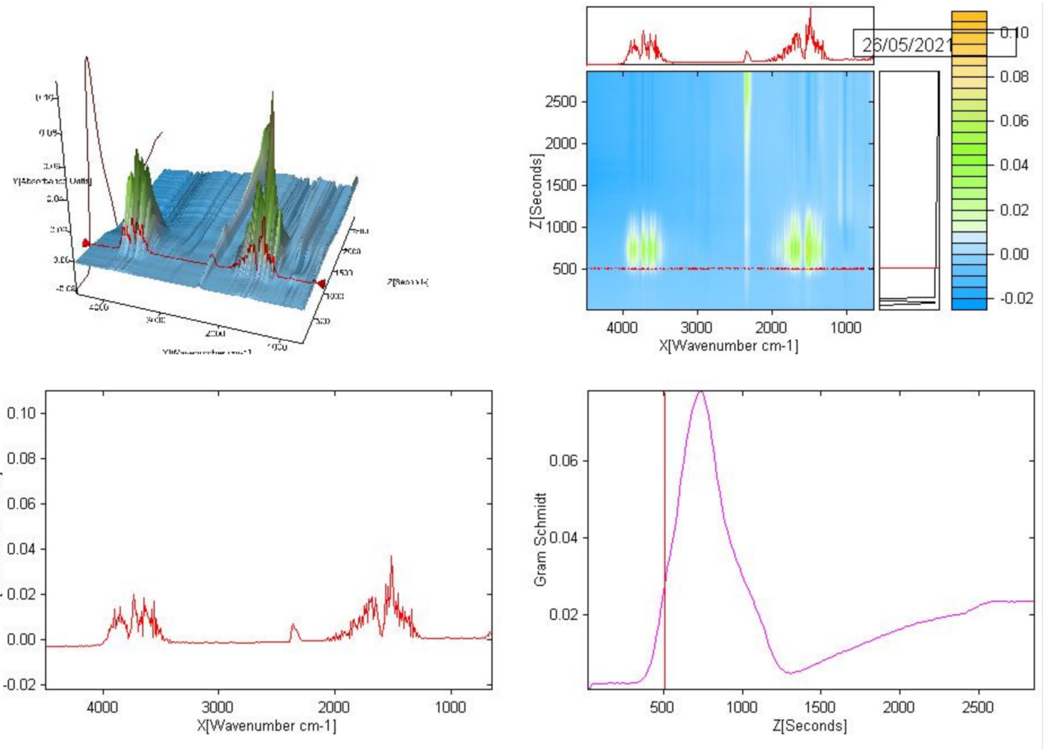

Figure S10. The TG-FTIR of 2-propen-1-ol oligomers obtained using [VOO(dipic)](2-phepyH) · H_2O
